# FMO4 shapes immuno‐metabolic reconfiguration in hepatocellular carcinoma

**DOI:** 10.1002/ctm2.740

**Published:** 2022-02-20

**Authors:** Yan Luo, An‐Na Chen, Jian‐Tao Fu, Gang Zhou, Jie Wang, Xiang Zhou, Jin Yang, Jun‐Ping Shi

**Affiliations:** ^1^ Department of Translational Medicine Center Affiliated Hospital of Hangzhou Normal University Hangzhou P. R. China; ^2^ Institute of Hepatology and Metabolic Diseases Hangzhou Normal University Hangzhou P. R. China

Dear Editor,

Hepatocellular carcinoma (HCC) is the fourth leading cause of cancer death worldwide. Currently, the overall response rate of immune checkpoint blockade (ICB) is only 15%–20% in HCC patients, suggesting the critical need to overcome this barrier via a comprehensive examination of the mechanisms underlying local and systemic anti‐tumour immune responses.^1^ Here, we explored the association between flavin mono‐oxygenase enzyme 4 (FMO4) and immuno‐metabolic signatures in HCC. Our results indicate that FMO4 may be a useful prognostic biomarker and therapeutic target for HCC.

FMO4 expression was decreased in tumour tissues relative to normal tissues in multiple HCC cohorts (Figure 1A). Overall survival (OS) and progression‐free interval were significantly reduced in FMO4^low^ tumours when using median value as cut‐off (Figure 1B). FMO4^low^ remained a significant predictive factor for survival in HCC patients after multivariate analysis (Figure S1C).

**FIGURE 1:**
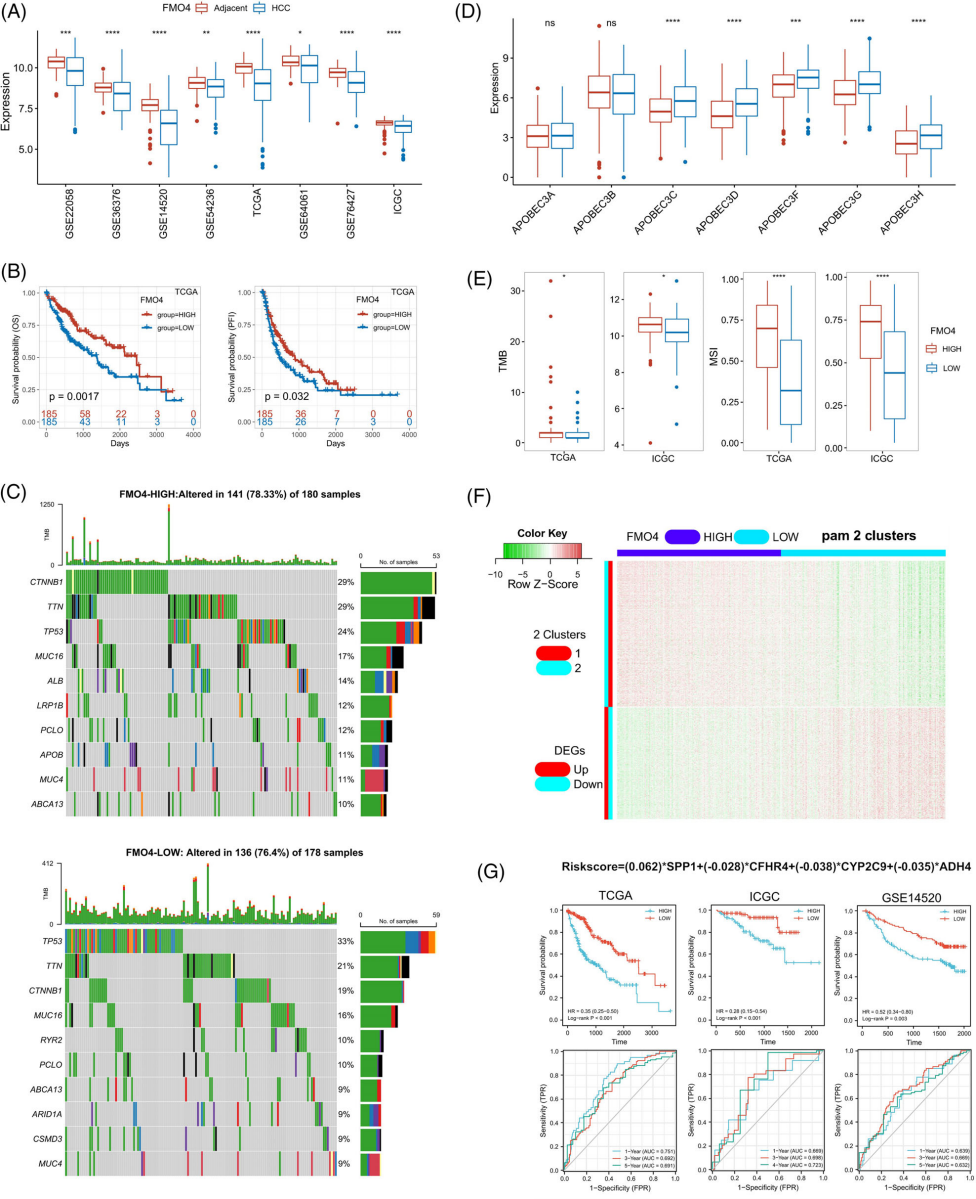
Characterization of flavin mono‐oxygenase enzyme 4 (FMO4) expression in hepatocellular carcinoma (HCC). (A) Eight HCC cohorts indicated the down‐regulation of FMO4 in tumour tissues. (B) FMO4 expression is associated with adverse outcome in The Cancer Genome Atlas (TCGA) cohort. (C) Profiles of HCC mutations in FMO4^low^ and FMO4^high^ groups. (D) Differences in individual APOBEC3 family gene expression between FMO4 groups. (E) Tumour burdern (TMB) and microsatellite instability (MSI) score between the two groups in TCGA or International Cancer Genome Consortium (ICGC) cohort, respectively. (F) Heatmap of co‐expressed profiles of HCC. The partitioning around medoids algorithm (PAM) is used to cluster the co‐expressed genes into two clusters. (G) FRS survival prediction accuracy in the various HCC datasets.

In addition, FMO4^low^ group's genomic landscape was substantially different from that of the FMO4^high^ group (Figure 1C, Figure S1F,G). FMO4^low^ HCCs were linked with increased expression of members of the APOBEC3 genes (Figure 1D), which contribute to cancer heterogeneity. A negative correlation between FMO4 and tumour burdern or microsatellite instability was observed, indicating that FMO4^low^ HCCs are more immunogenic^2^ (Figure 1E).

Next, co‐expression and cluster analysis clearly revealed two clusters of differentially expressed genes (DEGs) associated with FMO4 status (Figure 1F). Using the LASSO method to identify the top candidate DEGs (FMO4‐related signature, FRS; Figure S2F–H), patients with a low FRS had a substantially longer OS and survival probability in various HCC cohorts (Figure 1G, Tables S1 and S2).This re‐validated the conclusion that FMO4 was a prognostic biomarker in HCC.

Metabolic alterations are one of the hallmarks of cancer.^3^ We next investigated the configuration of the metabolic landscape according to FMO4 status in HCC (Table S3). The top positively correlated pathways were shown in Figure 2A. All these pathways were down‐regulated in the FMO4^low^ group and further associated with the adverse outcome (Figure 2B,C). Of note, drug induction of bile acid (BA) showed the highest correlation with FMO4 expression (Figure 2D,E). Once known exclusively for their role in nutrient absorption, BAs are now recognized as signaling molecules to be linked to inflammation and immunity. As expected, BA‐high tumours have significantly improved the prognosis in various HCC cohorts (Figure 2F).

**FIGURE 2:**
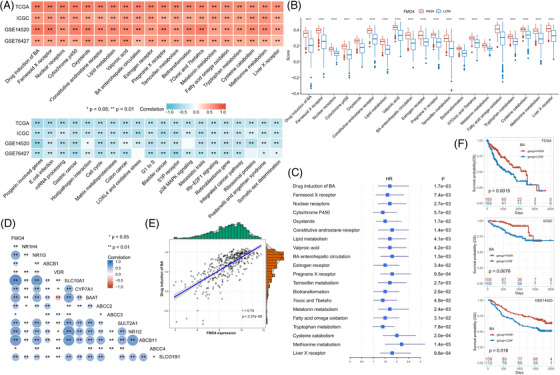
FMO4 alteration is associated with metabolic reconfiguration. (A) Top 20 positive and negative correlations between FMO4 and metabolic pathways, using multiple cohorts to validate. (B) Top 10 positively correlated metabolic pathways were differential configured between FMO4^low^ and FMO4^high^ state. (C) A forest plot showing the activity of the top 20 positively linked metabolic pathways by univariate cox analysis. (D) The expression of the genes responsible for bile acid (BA) pathway was significantly correlated with FMO4 expression. (E) Correlation of FMO4 with the BA pathway. (F) Low activity of BA metabolism implies adverse outcome in multiple HCC cohorts.

A wide range of immunomodulators were shown to be adversely associated with FMO4 (Figure 3A). For instance, the chemokines including CCL20/CXCR3 recruiting regulatory T cells (Treg) and CXCL1/CXCR2 attracting myeloid‐derived suppressor cell (MDSC) were elevated in FMO4^low^ HCCs (Figure S3A,B).

**FIGURE 3:**
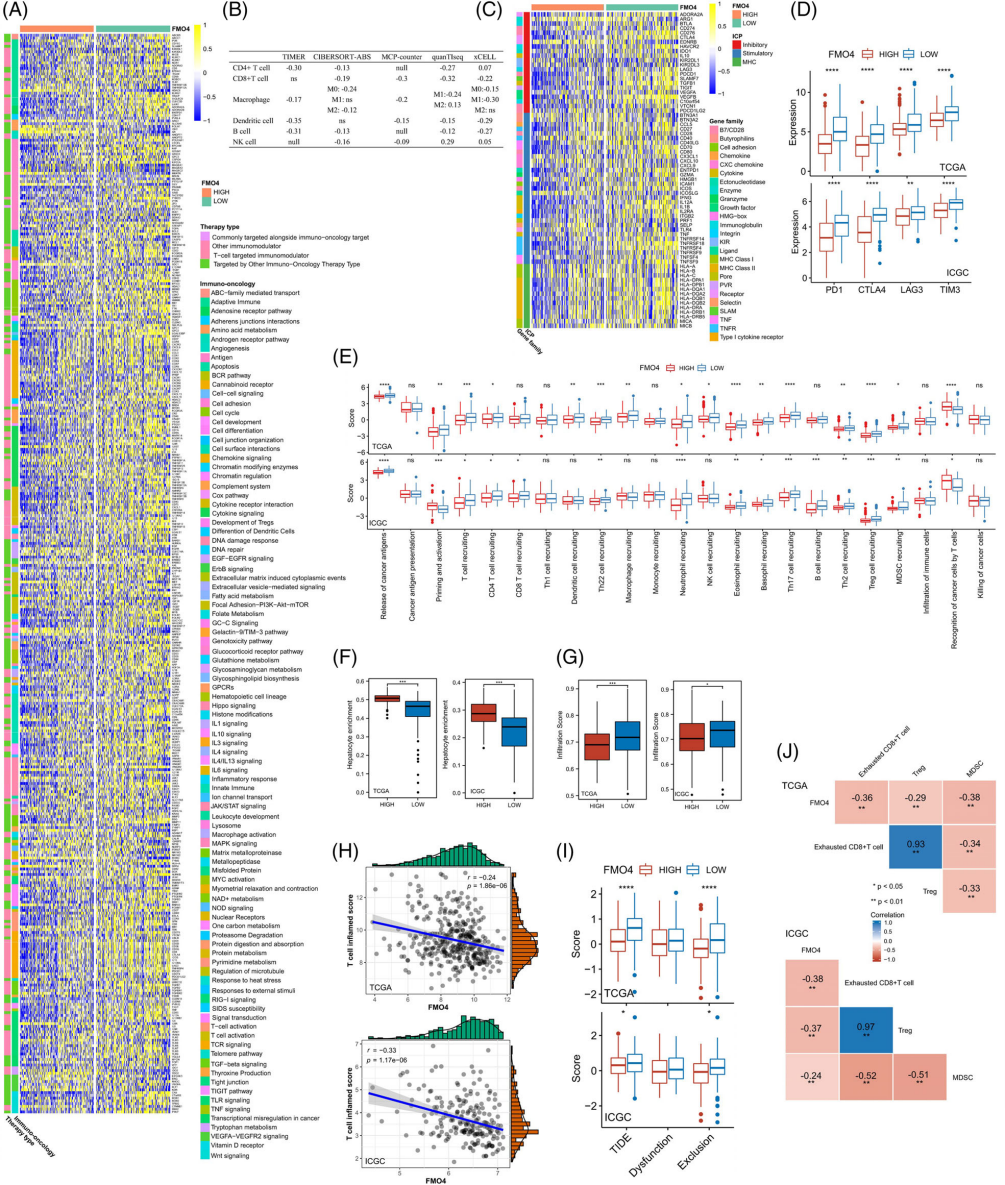
FMO4 alteration forms a unique tumour microenvironment (TME) in HCC. (A) Differences in immunomodulator (chemokines, cytokines, receptors, MHC, immunostimulators and immunoinhibitors) expression between FMO4^high^ and FMO4^low^ groups in HCC. (B) Correlation between FMO4 and the infiltration levels of six types of tumour‐infiltrating immune cells (TIICs) (CD4^+^ T cells, CD8^+^ T cells, NK cells, Macrophages, B cells and Dendritic cells), as determined by five distinct algorithms. (C) Differences in the expression of immune checkpoint‐related genes between FMO4^high^ and FMO4^low^ states. (D) Association between the FMO4 and major T cell exhaustion marker genes. (E) Differences between FMO4^high^ and FMO4^low^ groups at each stage of the cancer immunity cycle. (F and G) Box plot showing the distribution of hepatocytes, and infiltration score between FMO4^high^ and FMO4^low^ states, respectively. (H and I) Correlations between FMO4 and T cell inflamed score (TIS) or tumour immune dysfunction and exclusion (TIDE) score, respectively. (J) Correlation between FMO4 expression and exhausted CD8^+^ T cell activity, Treg activity and MDSC activity.

To characterize the tumour microenvironment (TME), tumour‐infiltrating immune cells were estimated by TIMER, CIBERSORT, quanTIseq,MCP‐counter and xCell algorithms.^4^ FMO4^low^ tumours have a significantly high infiltration of anticancer immune cells, such as activated CD8^+^ or CD4^+^ T cells and M1 macrophages. FMO4^low^ tumours also have a high infiltration of procancer immune cells, such as neutrophil, MDSC, M2 macrophage, and Treg; and simultaneously have the loss of hepatocytes (Figure 3B,F; Figure S3). As a consequence, the bulk of the processes in the cancer immunity cycle was shown to be elevated in the FMO4^low^ group (Figure 3E).

Immune checkpoint inhibitor expression was shown to be low in non‐inflamed TME. FMO4 was consistently observed to have a negative correlation with the majority of immune checkpoint inhibitors, including PD1, CTLA4, LAG3 and TIM3 (Figure 3C,D).

T cell inflamed score (TIS) is a marker evaluating pre‐existing but suppressed adaptive immunity.^5^ As expected, FMO4 was inversely linked with the TIS score (Figure 3H). Further, tumour immune dysfunction and exclusion (TIDE) score^6^ (especially exclusion score) was elevated in the FMO4^low^ group, which implies the ICB resistance (Figure 3I). Mechanistically, FMO4^low^ status was evidently correlated with the enrichment of exhausted CD8^+^ T cells, MDSCs and Tregs, and the immunotherapy‐related pathways (Figures 3J and 4A). Thus, FMO4^low^ is closely associated with the formation of an inflamed yet exhausted TME.

**FIGURE 4:**
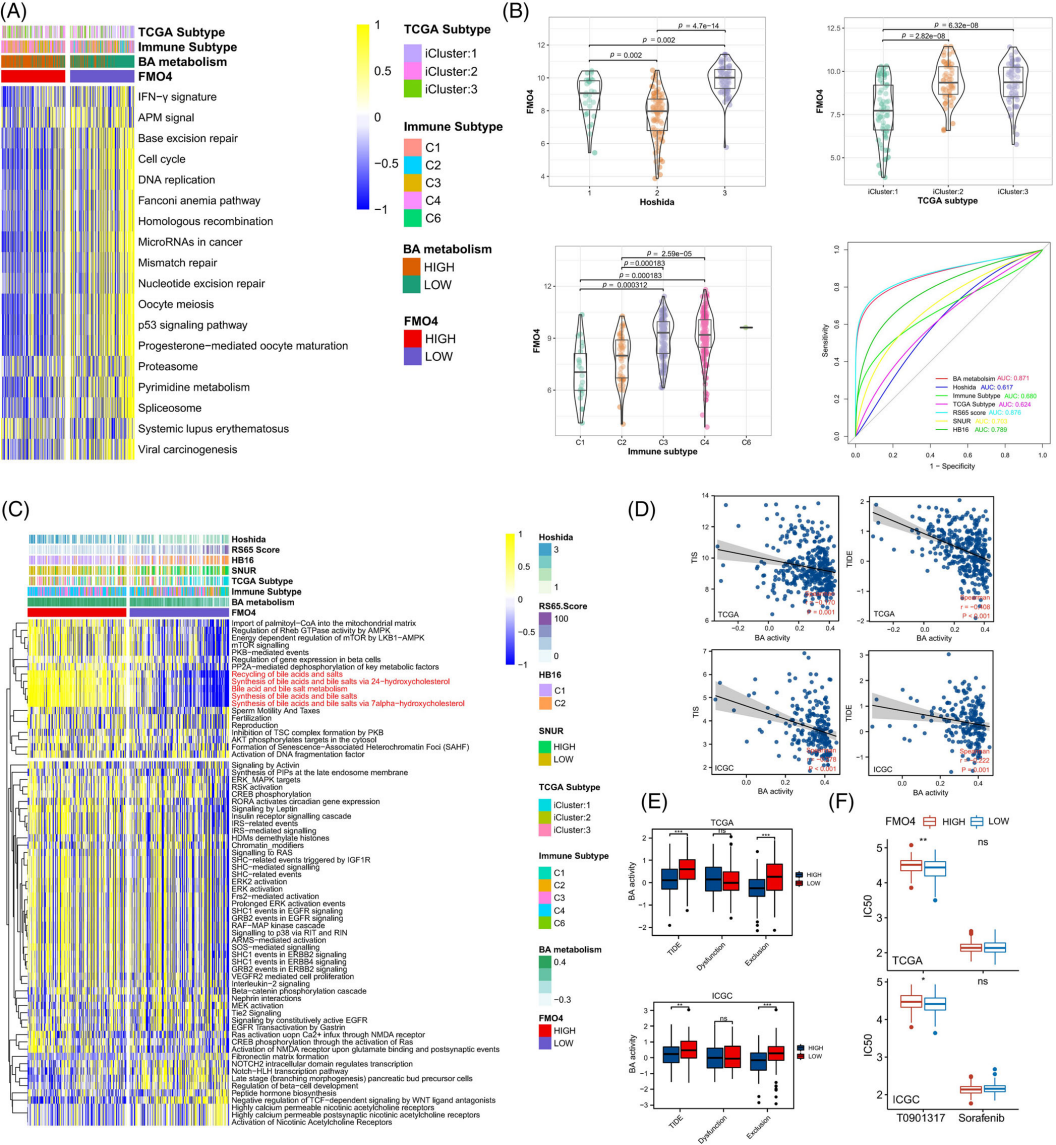
FMO4 predicts the molecular subtype and the therapeutic option. (A) Differences in the enrichment scores of immunotherapy‐related pathways between FMO4^high^ and FMO4^low^ groups. (B) FMO4 expression in the main HCC subtypes. Predictive accuracy of FMO4 for molecular subtypes using seven different typing algorithms. (C) Correlations between FMO4 and HCC‐specific pathway signatures. (D) Correlations between bile acid (BA) activity and TIS or TIDE score, respectively. (E) Differences in TIDE score between BA^high^ and BA^low^ groups. (F) IC50 of T0901317 and sorafenib in TCGA and ICGC cohort, respectively.

Previous studies elucidated several molecular subtypes of HCC.^7^, ^8^, ^9^ Indeed, FMO4 expression was significantly lowly expressed in iClust1^7^ and C1^8^ group, respectively (Figure 4B). Additionally, among the seven subtyping systems, FMO4^low^ HCC was more likely to be the RS65 subtype (Figure 4B). In fact, genes associated with normal metabolic functions of liver are enriched in low risk genes within RS65 system.^9^ HCC‐specific pathway analysis re‐validated the down‐regulation of BA activity in FMO4^low^ group (Figure 4C). Association with the TIS, and TIDE between FMO4 and BA activity showed the similar pattern (Figure 4D,E). Since T0901317 is a dual LXR/FXR agonist to promote BA metabolism,^10^ we showed that FMO4^low^ was associated with a lower IC50 of T0901317 in both TCGA and ICGC cohort (Figure 4F). Therefore, restoring BA signaling by correcting FMO4 dysregulation may be helpful for the treatment of HCC with low FMO4 expression.

Due to the immune tolerance in liver microenvironment, liver cancer is still among the most difficult‐to‐treat human cancers. Cellular metabolism has emerged as a critical determinant of the function of both cancer and immune cells. Manipulating the metabolic pathways in therapeutically meaningful ways can overcome T cell dysfunction and reconfigure the metabolic balance in the TME. Indeed, FMO4 expression is closely associated with the activity of BA metabolism and down‐regulated in the FMO4^low^ condition. To enhance immunotherapy response, the combination of immunotherapy with restoring BA activity such as T0901317 might help raise the efficacy of ICB in HCC.

In summary, this study confirmed that low expression of FMO4 is an adverse biomarker for HCC. FMO4‐related BA metabolism is critical for the efficacy of immune response in HCC.

## CONFLICT OF INTEREST

The authors declare that they have no competing interests.

## Supporting information

SUPPORTING INFORMATION

SUPPORTING INFORMATION
