# Supplementary material for: FMO4 shapes immuno‐metabolic reconfiguration in hepatocellular carcinoma
Source: Clin Transl Med. 2022 Feb 20;12(2):e740. doi: 10.1002/ctm2.740 (PMC8858612; doi:10.1002/ctm2.740)
Supplement: Supplementary file 1 — SUPPORTING INFORMATION [file CTM2-12-e740-s001.docx]

**Online Methods**

**Patient cohort and data processing**

Clinical, genomic, and transcriptome data were acquired from the TCGA liver hepatocellular carcinoma (LIHC) cohort from the GDC legacy archive, as well as the International Cancer Genome Consortium (ICGC, https://www.icgc-argo.org). TPM values were used to calculate mRNA expression levels. HCC microarray datasets such as GSE14520 ^1^, GSE36376, GSE25007, GSE76427 and GSE9843, *etc*, were obtained from GEO ([http://www.ncbi.nlm.nih.gov/geo](http://www.ncbi.nlm.nih.gov/geo)). The raw data were normalised and non-expressed and non-informative genes were filtered.

**Co-expression analysis and functional annotation**

Co-expressed gene-set enrichment analysis (cogena) was used to illuminate coordinated changes accompanying FMO4 alteration ^2^. Gene set enrichment analysis (GSEA) was used to compare the FMO4^low^ (186 patients) and FMO4^high^ (185 patients) groups using annotated Kyoto Encyclopedia of Genes and Genomes (KEGG) gene sets. The GSEA's main output is the enrichment score (ES) and normalized enrichment score (NES), which indicate whether a particular gene set is boosted at the top or bottom of the list of genes. The metabolic profile of HCC was quantified using the single sample gene set enrichment analysis (ssGSEA) technique based on MSigDB gene sets ^3^.

**Estimation of tumor immune-related features**

T cell inflamed score (TIS), defining pre-existing cancer immunity, was computed as a weighted linear combination of the scores from the 18 genes ^4^. A list of immunomodulatory genes including chemokines, cytokines, receptors, MHC and immune stimulators, inhibitory immune checkpoints was curated from the previous study ^5^.

**Characteristics of the tumor microenvironment (TME) in HCC**

Immunological features of the TME in HCC include tumor-infiltrating immune cells (TIICs) infiltration, cancer immunity cycle activity, and expression of immune checkpoint inhibitors. We estimated the amount of TIICs in a comprehensive manner using five different algorithms: Cibersort ^6^, MCP-counter, quanTIseq, TIMER, and xCell ^7^. The cancer immunity cycle reflects the anticancer immune response and consists of seven steps: antigen release from cancer cells (Step 1), antigen presentation from cancer cells (Step 2), priming and activation of immune cells (Step 3), immune cell trafficking to tumors (Step 4), immune cell infiltration into tumors (Step 5), recognition of cancer cells by T cells (Step 6), and killing of cancer cells (Step 7) ^8, 9^. To assess the destiny of the tumor cells, the activities of these stages were examined using ssGSEA.

**Development of an FMO4-related risk score**

The limma package ^10^ was used to find differentially expressed genes (DEGs) with a false discovery rate (FDR) less than 0.05 and an absolute logFC more than 1 between the FMO4^low^ and FMO4^high^ groups. ClusterProfiler R software was used to conduct Gene Ontology (GO) and KEGG enrichment.

We used the survival package to perform univariate Cox analysis on common DEGs. Additionally, the least absolute shrinkage and selector operation (LASSO) method was used to screen for the most discriminative candidate DEGs. Then, using the multivariate Cox regression coefficient, we generated an FMO4-related risk score (FRS) based on the FRS RNA-expression profiles:

FRS=$\sum\beta i*RNAi$

where βi is the coefficient associated with the 'i'th FRS RNA-expression profile. Patients were divided into high and low FRS groups according to their median FRS. The Kaplan-Meier method and the log-rank test were used to compare the groups statistically in order to determine the FRS's prognostic relevance. The FRS's statistical performance was evaluated using the tROC R program.

**Treatment exploration**

We predicted the therapeutic response based on the pharmacogenomics database [Genomics of Drug Sensitivity in Cancer (GDSC), [https://www.cancerrxgene.org/].](https://www.cancerrxgene.org/%5d.) The prediction procedure was carried out using pRRophetic, in which the half-maximal inhibitory concentration (IC50) of the samples was determined using ridge regression and the prediction accuracy was calculated. The Wilcoxon signed-rank test was used to compare the IC50 values between the high and low risk groups. The tumor immune dysfunction and exclusion (TIDE) algorithm was used to predict potential ICB treatment response ^11^.

**Statistical Analysis**

Overall survival (OS) was defined as the time interval between diagnosis and death from any cause, while progression-free interval (PFI) was defined as the time period between diagnosis and advancement of HCC. Kaplan-Meier plots were created to depict the survival disparities. The Mann-Whitney test was used to determine the differences between the two groups for continuous variables and chi-square testing for categorical variables. A p-value of 0.05 on both sides was deemed statistically significant.

**Online references**

1. Roessler S, Jia HL, Budhu A, Forgues M, Ye QH, Lee JS, et al. A unique metastasis gene signature enables prediction of tumor relapse in early-stage hepatocellular carcinoma patients. Cancer research. 2010;**70**(24):10202-10212.

2. Jia Z, Liu Y, Guan N, Bo X, Luo Z, Barnes MR. Cogena, a novel tool for co-expressed gene-set enrichment analysis, applied to drug repositioning and drug mode of action discovery. BMC genomics. 2016;**17**:414.

3. Hänzelmann S, Castelo R, Guinney J. GSVA: gene set variation analysis for microarray and RNA-seq data. BMC Bioinformatics. 2013;**14**:7.

4. Danaher P, Warren S, Lu R, Samayoa J, Sullivan A, Pekker I, et al. Pan-cancer adaptive immune resistance as defined by the Tumor Inflammation Signature (TIS): results from The Cancer Genome Atlas (TCGA). J Immunother Cancer. 2018;**6**(1):63.

5. Eddy JA, Thorsson V, Lamb AE, Gibbs DL, Heimann C, Yu JX, et al. CRI iAtlas: an interactive portal for immuno-oncology research. F1000Res. 2020;**9**:1028.

6. Newman AM, Liu CL, Green MR, Gentles AJ, Feng W, Xu Y, et al. Robust enumeration of cell subsets from tissue expression profiles. Nat Methods. 2015;**12**(5):453-457.

7. Aran D, Hu Z, Butte AJ. xCell: digitally portraying the tissue cellular heterogeneity landscape. Genome Biol. 2017;**18**(1):220.

8. Chen DS, Mellman I. Oncology meets immunology: the cancer-immunity cycle. Immunity. 2013;**39**(1):1-10.

9. Xu L, Deng C, Pang B, Zhang X, Liu W, Liao G, et al. TIP: A Web Server for Resolving Tumor Immunophenotype Profiling. Cancer Res. 2018;**78**(23):6575-6580.

10. Ritchie ME, Phipson B, Wu D, Hu Y, Law CW, Shi W, et al. limma powers differential expression analyses for RNA-sequencing and microarray studies. Nucleic Acids Res. 2015;**43**(7):e47.

11. Jiang P, Gu S, Pan D, Fu J, Sahu A, Hu X, et al. Signatures of T cell dysfunction and exclusion predict cancer immunotherapy response. Nat Med. 2018;**24**(10):1550-1558.

**Supplementary Figure S1**

**
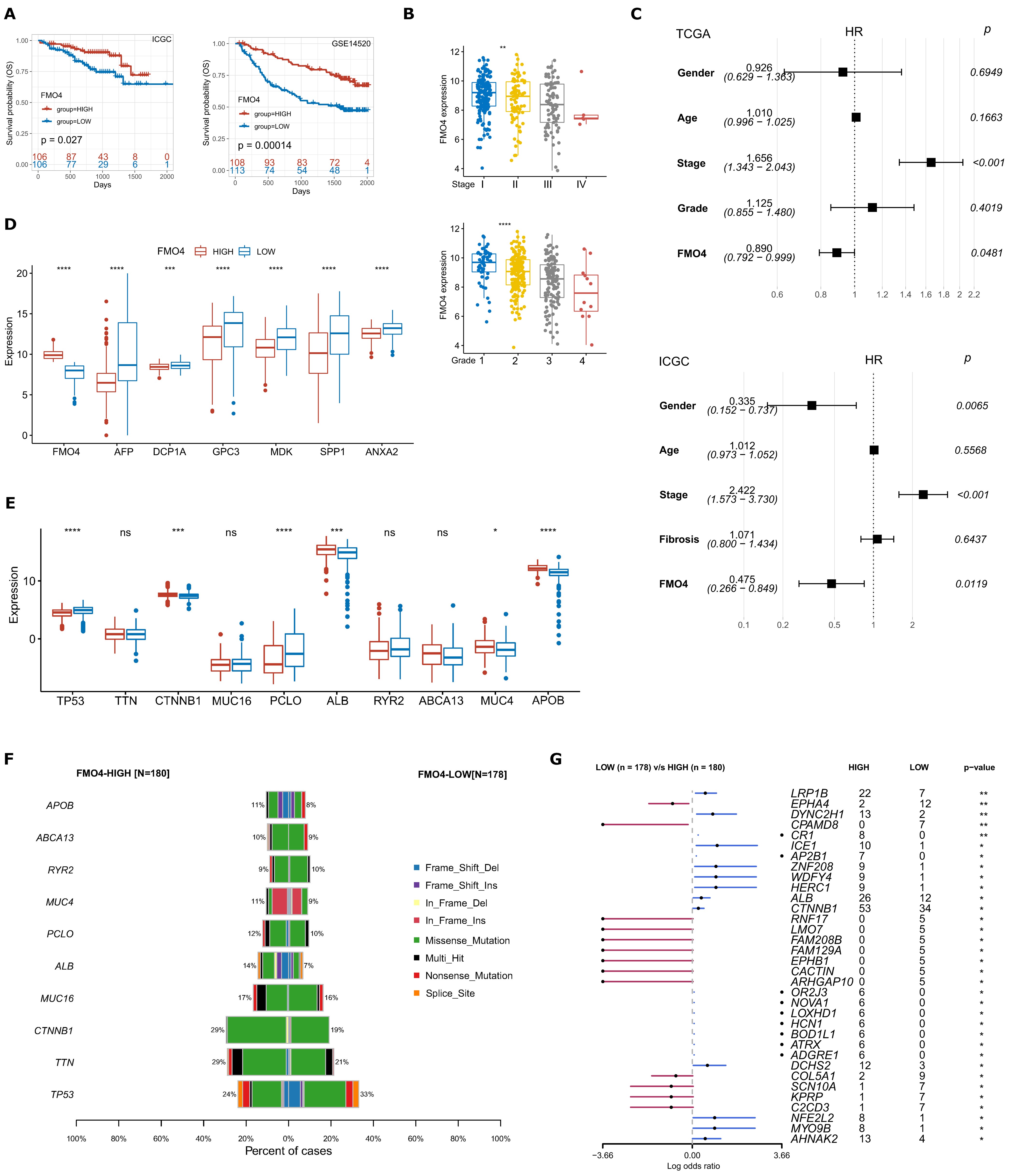
**

Characteristics of FMO4 expression and mutation pattern. (A) FMO4 down-regulation is associated with adverse outcome in ICGC and GSE14520 cohort, respectively. (B) FMO4 expression decreased along with the progression of tumor stage or grade. (C) Multivariate analysis demonstrates that FMO4 is an independent prognostic factor in HCC. (D) The FMO4^low^ group was more likely to have greater levels of well-characterized unfavorable prognostic biomarkers of HCC. (E) Expression differences in the top ten mutated genes between FMO4 groups. (F) coBarplot comparing genetic profile between FMO4^low^ and FMO4^high^ HCCs. (G) Prognosis of differentially mutated genes between FMO4^low^ and FMO4^high^ HCCs.

**Supplementary Figure S2**


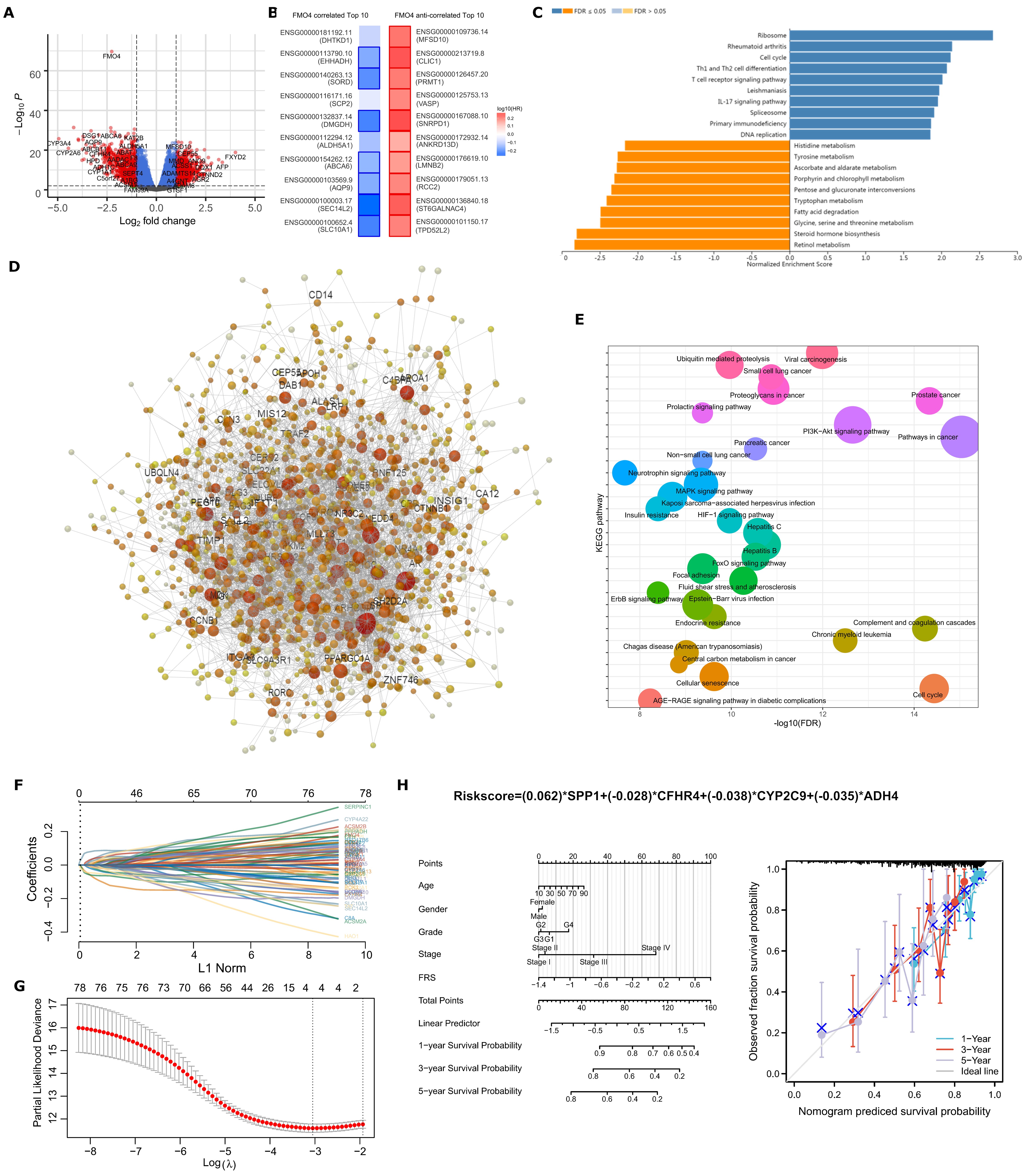


Genome-wide profile associated with FMO4 expression. (A) Volcano plot of differential expressed genes between FMO4^high^ and FMO4^low^ group. (B) Survival map of FMO4-correlated top 10 genes. The red and blue blocks denote higher and lower risks, respectively. The box denotes the significant result in prognostic analyses. (C) GSEA findings for the function change associated with FMO4-related DEGs. (D) Liver-specific FMO4-related protein-protein interaction network. The liver specific protein-protein interaction data were collected from the DifferentialNet database. (E) KEGG enrichment of the network (D). (F) LASSO coefficient profiles of 80 prognostic RNAs in TCGA cohort. The coefficient profile plot was developed against the log (Lambda) sequence. (G) Cross-validation for parameter selection in the LASSO regression model using minimal criteria. The FRS was developed using optimal RNAs with the highest discriminative capacity. (H) Nomogram to predict the 1-y，2-y and 3-y overall survival. The right panel calibration curve for the overall survival nomogram model.

**Supplementary Figure S3**


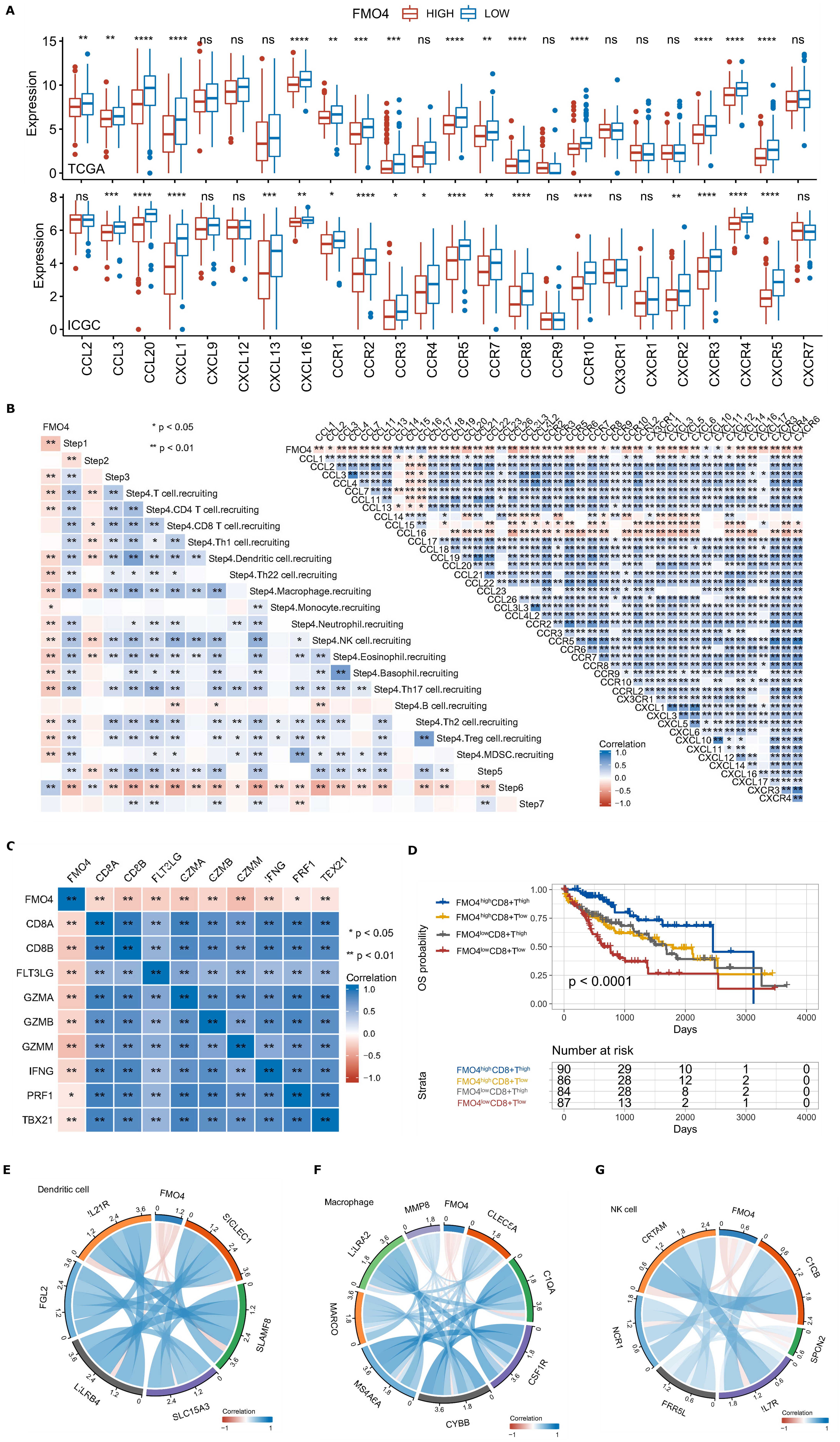


Immune characteristics of FMO4 expression in HCC. (A) Expression differences of chemokines between FMO4^high^ and FMO4^low^ groups. (B) Correlation between FMO4 and chemokines and cancer immunity cycle respectively. (C) Correlations between FMO4 and effector genes of CD8+ T cells. (D) Survival plot of FMO4 associated CD8+T cells. (E-G) Correlations between FMO4 and marker genes of dendritic, macrophage, and NK cells respectively.

**Supplementary Table S1**

Table 1 Multivariate analysis of FRS in TCGA cohort

| TCGA | Total(N) | Univariate analysis | |  | Multivariate analysis | |
| --- | --- | --- | --- | --- | --- | --- |
|  |  | Hazard ratio (95% CI) | P value |  | Hazard ratio (95% CI) | P value |
| Age | 366 | 1.012 (0.999-1.026) | 0.078 |  | 1.009 (0.995-1.023) | 0.226 |
| Gender | 366 |  |  |  |  |  |
| Male | 246 | Reference |  |  |  |  |
| Female | 120 | 1.225 (0.860-1.746) | 0.260 |  |  |  |
| Grade | 361 |  |  |  |  |  |
| G1 | 55 | Reference |  |  |  |  |
| G2 | 175 | 1.179 (0.696-1.997) | 0.541 |  |  |  |
| G3 | 119 | 1.232 (0.710-2.139) | 0.457 |  |  |  |
| G4 | 12 | 1.692 (0.625-4.580) | 0.301 |  |  |  |
| Stage | 342 |  |  |  |  |  |
| Stage I | 170 | Reference |  |  |  |  |
| Stage II | 84 | 1.423 (0.872-2.323) | 0.158 |  | 1.050 (0.631-1.747) | 0.851 |
| Stage III | 83 | 2.676 (1.754-4.083) | **<0.001** |  | 2.119 (1.363-3.293) | **<0.001** |
| Stage IV | 5 | 5.496 (1.695-17.821) | **0.005** |  | 5.163 (1.592-16.741) | **0.006** |
| FRS | 366 | 3.582 (2.370-5.412) | **<0.001** |  | 2.996 (1.876-4.785) | **<0.001** |

**Supplementary Table S2**

Table 2 Multivariate analysis of FRS in ICGC cohort

| ICGC | Total(N) | Univariate analysis | |  | Multivariate analysis | |
| --- | --- | --- | --- | --- | --- | --- |
|  |  | Hazard ratio (95% CI) | P value |  | Hazard ratio (95% CI) | P value |
| Age | 212 | 1.015 (0.980-1.052) | 0.406 |  |  |  |
| Gender | 212 |  |  |  |  |  |
| Male | 162 | Reference |  |  |  |  |
| Female | 50 | 1.938 (0.962-3.905) | 0.064 |  | 2.570 (1.179-5.602) | **0.018** |
| Stage | 212 |  |  |  |  |  |
| I | 33 | Reference |  |  |  |  |
| II | 102 | 2.372 (0.539-10.443) | 0.254 |  | 2.011 (0.454-8.903) | 0.357 |
| III | 61 | 4.289 (0.965-19.065) | 0.056 |  | 5.058 (1.100-23.248) | **0.037** |
| IV | 16 | 12.022 (2.488-58.092) | **0.002** |  | 8.779 (1.677-45.965) | **0.010** |
| FRS | 212 | 4.701 (2.575-8.584) | **<0.001** |  | 3.506 (1.905-6.452) | **<0.001** |
